# Novel Formulation of Undecylenic Acid induces Tumor Cell Apoptosis

**DOI:** 10.3390/ijms232214170

**Published:** 2022-11-16

**Authors:** Zoe I. Day, Alyce J. Mayfosh, Marie-Claire Giel, Yuning Hong, Scott A. Williams, Jascinta P. Santavanond, Thomas F. Rau, Ivan K. Poon, Mark D. Hulett

**Affiliations:** 1 Department of Biochemistry and Chemistry, School of Agriculture, Biomedicine and Environment, La Trobe Institute for Molecular Science, La Trobe University, Bundoora, VIC 3086, Australia; 2 Wintermute Biomedical, 789 Bauer Lane, Corvallis, MT 59828, USA

**Keywords:** undecylenic acid, anticancer, cancer, apoptosis, fatty acid, monounsaturated fatty acid, lipid droplets

## Abstract

Undecylenic acid, a monounsaturated fatty acid, is currently in clinical use as a topical antifungal agent, however the potential for therapeutic application in other disease settings has not been investigated. In this study, we describe a novel platform for the solubilization of fatty acids using amino acids and utilize this approach to define a tumoricidal activity and underlying mechanism for undecylenic acid. We examined a novel formulation of undecylenic acid compounded with L-Arginine, called GS-1, that induced concentration-dependent tumor cell death, with undecylenic acid being the cytotoxic component. Further investigation revealed that GS-1-mediated cell death was caspase-dependent with a reduction in mitochondrial membrane potential, suggesting a pro-apoptotic mechanism of action. Additionally, GS-1 was found to localize intracellularly to lipid droplets. In contrast to previous studies where lipid droplets have been shown to be protective against fatty acid-induced cell death, we showed that lipid droplets could not protect against GS-1-induced cytotoxicity. We also found a role for Fatty Acid Transport Protein 2 (FATP2) in the uptake of this compound. Collectively, this study demonstrates that GS-1 has effective pro-apoptotic antitumor activity in vitro and, together with the novel platform of fatty acid solubilization, contributes to the re-emerging field of fatty acids as potential anti-cancer therapeutics.

## 1. Introduction

Undecylenic acid is an 11-carbon monounsaturated fatty acid (MUFA) derived from the distillation of castor oil via pyrolysis [1]. Undecylenic acid is also naturally found in human sweat. Currently, undecylenic acid is only known for its antifungal properties and is the active ingredient for many topical antifungal treatments [2,3]. The antifungal effects of undecylenic acid have been extensively investigated in *Candida albicans* where studies have shown that undecylenic acid can inhibit biofilm formation. At concentrations above 3 mM genes related to hyphal formation, such as hyphal wall protein 1 (HWP1), are downregulated at the transcriptional level leading to poor biofilm formation, a step that is important during skin infection [3]. Outside this, little is known about this fatty acid, including whether it has other potential therapeutic applications such as in a cancer setting.

The anti-cancer properties of several fatty acids have previously been demonstrated against several cancer cell lines, including gastric [4], colon [5], colorectal [6], breast [7], and cervical cancers [6,8] as well as in sarcoma [9], myeloma [10], and leukemia [11,12]. Some reports suggest that fatty acids have the potential to be selectively toxic to tumor cells, making them promising candidates for anti-cancer therapeutics [13,14,15]. For polyunsaturated fatty acids (PUFAs) it has been suggested that exogenous treatment of tumor cells induces oxidative stress and cancer cell death via augmentation of free radical levels and lipid peroxidation [8,10,11,12,13,14,16]. For example, docosahexaenoic acid and docosapentaenoic acid have been shown to induce lipid-peroxide-induced ferroptosis in HCT-116 colon cancer cell lines [6].

Several studies have also investigated the cytotoxic nature of saturated fatty acids (SFAs), like palmitate, and found that unlike PUFAs these fatty acids can induce cancer cell death via mitochondrial dysfunction through an Unfolded Protein Response (UPR) or diminution of the cardiolipin lipid fraction [7,17]. Furthermore, a PUFA-rich diet has been shown to delay tumor growth in an in vivo mouse xenograft model of colon cancer [6]. Therefore, while it is well understood that fatty acids can suppress the growth of tumor cells in vitro and in vivo, the molecular mechanism of their cytotoxicity has only been defined in select settings with apoptosis, autophagy, and ferroptosis all suggested as potential cell death pathways [18]. However, fewer studies have been conducted exploring the anti-cancer nature of monounsaturated fatty acids (MUFAs) such as undecylenic acid. Therefore, it is worthwhile investigating the potential cytotoxic roles of undecylenic acid against cancer cells.

The work presented in this paper provides some of the first evidence of the anti-cancer properties of undecylenic acid. GS-1, a novel formulation of undecylenic acid and L-arginine, presents a new strategy for the solubilization of fatty acids to be used in an anti-cancer setting. In solution, the carboxylic acid head group of undecylenic acid forms hydrogen bonds with the amine group of L-arginine allowing the non-polar lipid tails to pack together leaving the hydrogen-bonded amino acid on the outer surface to interact with water molecules and allow solubility in water. The novel formulation of undecylenic acid, GS-1, induces apoptotic cancer cell death with undecylenic acid being the cytotoxic component. The data suggest that GS-1-mediated cell death was caspase-dependent and resulted in typical apoptotic cell morphologies and a reduction in mitochondrial membrane potential. Like other fatty acids in previous studies, we found that GS-1 localized to lipid droplets after entering tumor cells via Fatty Acid Transport Protein 2 (FATP2). While lipid droplets do not play a protective role against GS-1-induced cell death, we were able to determine that GS-1 likely enters cells prior to mediating cell death. These data demonstrate that GS-1 has effective antitumor activity in vitro and lays the foundation for exploring other novel applications for undecylenic acid.

## 2. Results

### 2.1. Biophysical Properties of GS-1

**Figure 1:**
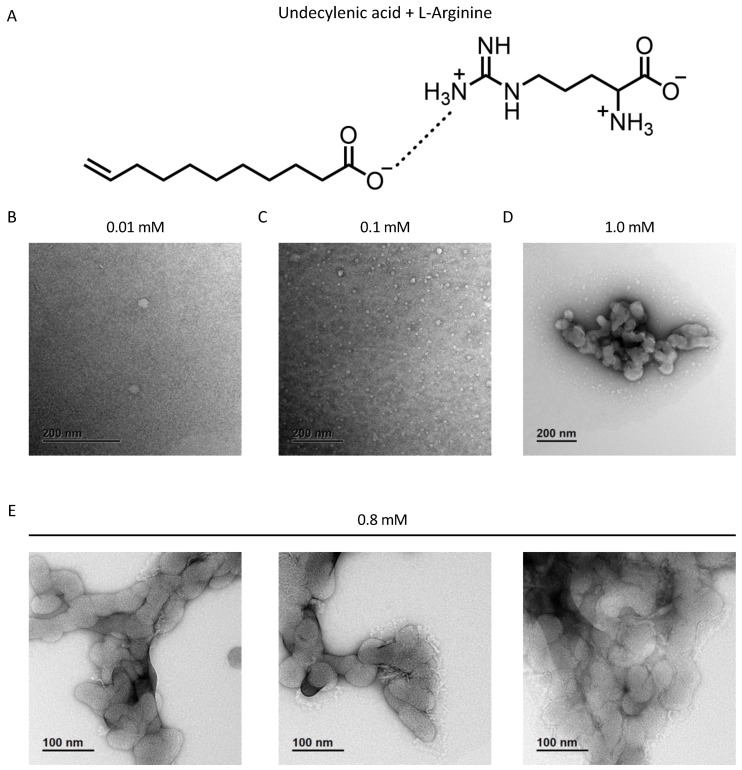
Biophysical characterization of GS-1. (**A**) Schematic representation of the structure and interactions of GS-1 components. (**B**–**E**) TEM of GS-1 was performed at (**B**) 0.10 mM (150,00×), (**C**) 0.10 mM (100,000×), (**D**) 1.0 mM (80,000×), and (**E**) 0.80 mM (200,000×).

Fatty acids are naturally insoluble in water, requiring organic solvents or chemical modifications for utility. Since these solvents and modifications can hamper fatty acid activity [19,20], we sought to find a way to solubilize undecylenic acid, an 11-carbon monounsaturated fatty acid, in water to test its anticancer properties. Trials with ingredients that are Generally Regarded As Safe and Effective revealed that in the presence of L-arginine, undecylenic acid was completely soluble in water at concentrations exceeding 150 mg/mL at 20 °C. Based on the known chemical interactions between functional groups of these molecules and synthesis conditions employed here, upon compounding the basic amine group of L-arginine deprotonates the carboxylic group of undecylenic acid to form a highly stable ammonium carboxylate salt that is soluble in water (Figure 1A). This combination of undecylenic acid and L-arginine in water was given the name GS-1.

It was hypothesized that individual units of this arginine-undecylenic acid salt may form supramolecular structures in solution to shield the hydrophobic tail of the fatty acid from the polar water molecules. Indeed, TEM revealed that in solution GS-1 at 0.01–0.1 mM formed vesicle-like structures approximately 10–30 nm in diameter (Figure 1B,C). At concentrations above 0.8 mM, GS-1 formed tubular structures approximately 50 nm in diameter (Figure 1D,E). We propose that these structures are formed to shield the non-polar tail of undecylenic acid from water molecules in solution.

To further characterize the GS-1 formulation and confirm that no other products were produced upon compounding, GS-1 was separated into an aqueous fraction and a heptane fraction and subjected to 1H and 13C NMR. Spectrums detected in the aqueous and heptane fractions of GS-1 were consistent with the number of protons and carbons present in L-arginine (Supplementary Figure S1) and undecylenic acid (Supplementary Figure S2), respectively, agreeing with published work [21,22,23] GCMS analysis of aqueous and heptane fractions of GS-1 was also performed to detect any low concentrations of secondary products. As observed with NMR, only L-arginine (or the thermal decomposition structure of arginine) was detected in the aqueous fraction and only undecylenic acid was detected in the heptane fraction (Supplementary Figure S3). These results show there was no evidence of by-product formation, confirming that undecylenic acid and L-arginine interact non-covalently in solution and that no new compounds are formed upon combining.

### 2.2. GS-1 Is Cytotoxic to a Range of Tumor Cell Lines

**Figure 2:**
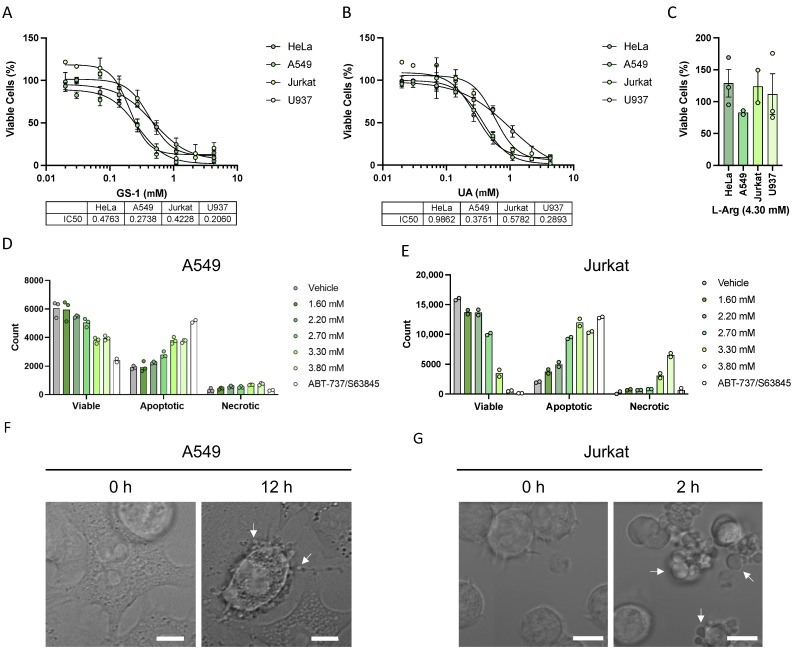
GS-1 induces apoptosis in tumor cells in vitro. HeLa, A549, Jurkat, and U937 cells were treated with increasing concentrations of (**A**) GS-1, (**B**) UA, or (**C**) 4.3 mM L-Arg for 24 h before undergoing MTT or MTS assays to determine viability as a percentage of vehicle controls (n = 2–3, mean ± S.E.M.). A549 cells were treated with GS-1 for 6 h and (**D**) Jurkat cells for 3 h (**E**) before flow cytometry analysis with Annexin V and To-Pro-3 to determine viable, apoptotic, and necrotic cell populations (representative of n = 3 experiments, mean). Bright field imaging of (**F**) A549 cells treated with GS-1 at 2.7 mM and (**G**) Jurkat cells treated with GS-1 at 2 mM (scale bars 10 µm; images representative of 3 experiments).

Initially, the cytotoxicity of GS-1 was tested against the human tumor cell lines HeLa, A549, Jurkat, and U937, where a dose-dependent reduction in viability was observed after 24 h of treatment (Figure 2A). Further, the cytotoxicity of GS-1 was tested against the primary Human Umbilical Vein Endothelial Cells (HUVEC) and a reduction in viability was also observed, suggesting a non-tumor cell specific mode of action in vitro (Supplementary Figure S4). Additional viability assays were performed using undecylenic acid and L-arginine individually. Assays with undecylenic acid alone demonstrated a similar dose-dependent reduction in viability to GS-1 itself (Figure 2B), whereas treatment with L-arginine had little effect on the viability of these same cell lines (Figure 2C), indicating that undecylenic acid confers GS-1 cytotoxicity.

To use a different method of assessing cell viability, flow cytometry was used to specifically look at the induction of apoptosis with Jurkat and A549 selected as representative cell lines for further experiments [24]. GS-1 induced a dose-dependent decrease in To-Pro-3^low^Annexin V (AV)^−^ cells (Viable) and a corresponding increase in To-Pro-3^intermediate^AV^+^ cells (Apoptotic) with little changes to To-Pro-3^high^AV^+^ cells (Necrotic) (Figure 2D,E) indicating phosphatidylserine exposure with some membrane permeabilization. These data demonstrate a potential pro-apoptotic activity for GS-1 in both A549 and Jurkat cell lines, and confirm that the reduced viability observed in Figure 2A does indeed correlate with cell death. Additionally, bright field microscopy also demonstrated that GS-1 treated A549 cells (Figure 2F) and Jurkat cells (Figure 2G) underwent membrane blebbing and cell disassembly characteristic of apoptotic cell death [25,26].

### 2.3. GS-1 Is Well Tolerated In Vivo

We also sought to determine the in vivo tolerability of GS-1 and whether there would be any toxic effects. A single ascending dose study was performed in which Sprague Dawley Rats were subcutaneously challenged with either 10 mL/kg saline or 10 mL/kg GS-1 at 190.5 mg/kg (at 19.05 mg/mL), 381 mg/kg (at 38.1 mg/mL), or 762 mg/kg (at 76.2 mg/mL). Rats that were given 190.5 mg/kg GS-1 exhibited no signs of toxicity or change in behaviour, suggesting this dose was well tolerated in vivo. Rats that were given 381 mg/kg or 762 mg/kg GS-1 displayed abnormal behaviour and toxicities within 30 min of receiving the dose, suggesting these doses exceed safe and tolerable limits. The safe dose achieved here with 190.5 mg/kg is extremely high, and likely exceeds any therapeutic exposure. Together, these results demonstrate that GS-1 was safe and well tolerated in vivo at a dose as high as 190.5 mg/kg.

### 2.4. GS-1 Induces Apoptosis in Tumor Cells

**Figure 3:**
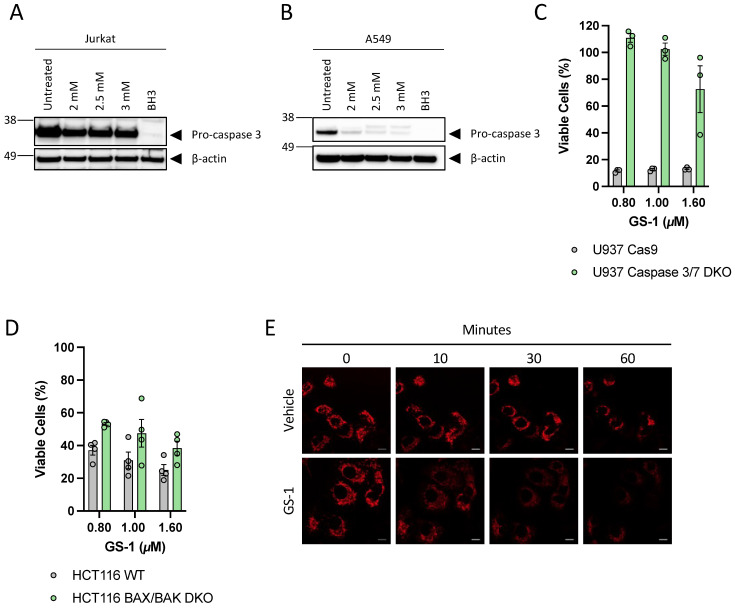
GS-1 toxicity relies on pro-apoptotic proteins. (**A**) Jurkat and (**B**) A549 cells were treated with increasing concentrations of GS-1 for 6 h before immunoblotting to determine pro-caspase 3 levels (representative of n = 3 independent experiments). (**C**) U937 WT and Caspase 3/7 DKO cells were treated with increasing concentrations of GS-1 before undergoing an MTS assay to determine viability (n = 3, ±S.E.M.). (**D**) HCT-166 WT and Bax/Bak DKO cells were treated with increasing concentrations of GS-1 before undergoing an MTT assay to determine viability (n = 3, ±S.E.M.). (**E**) Confocal time course microscopy analysis of A549 cells stained with MitoTracker Red and treated with GS-1 to determine change in mitochondrial membrane potential (scale bar 10 µm; images representative of 3 experiments).

To confirm the pro-apoptotic nature of GS-1-induced cell death we initially looked at the involvement of caspase 3 and 7, both well-established markers of apoptotic cell death. Immunoblotting assays demonstrated that GS-1 treatment for 6 h induced a modest reduction in pro-caspase-3 in Jurkat cells (Figure 3A), whereas a complete loss of pro-caspase-3 was observed in A549 cells after 6 h of treatment (Figure 3B). Moderate levels of cleaved caspase-3 were also detected in Jurkat cells after GS-1 treatment (Supplementary Figure S5). Confirming the involvement of caspases in GS-1 toxicity, caspase 3/7 double knock-out (DKO) cells [27] were also almost entirely protected from GS-1-mediated cell death compared to Cas9 controls (Figure 3C).

Further studies were conducted to investigate the role of other pro-apoptotic proteins such as Bax and Bak in GS-1-induced toxicity. The absence of Bax and Bak in DKO cells [28] provided modest protection from GS-1-induced cell death (Figure 3D). Since Bax and Bak are involved in the permeabilization of the mitochondrial membrane to release cytochrome C during intrinsic apoptosis, confocal microscopy was used to determine the effect of GS-1 on mitochondrial membrane potential. Consistent with apoptotic cell death GS-1-treated cells demonstrated a loss of mitochondrial membrane potential within 10 min of treatment (Figure 3E). These data suggest GS-1 is pro-apoptotic.

### 2.5. GS-1 Enters Cells to Induce Apoptosis

**Figure 4:**
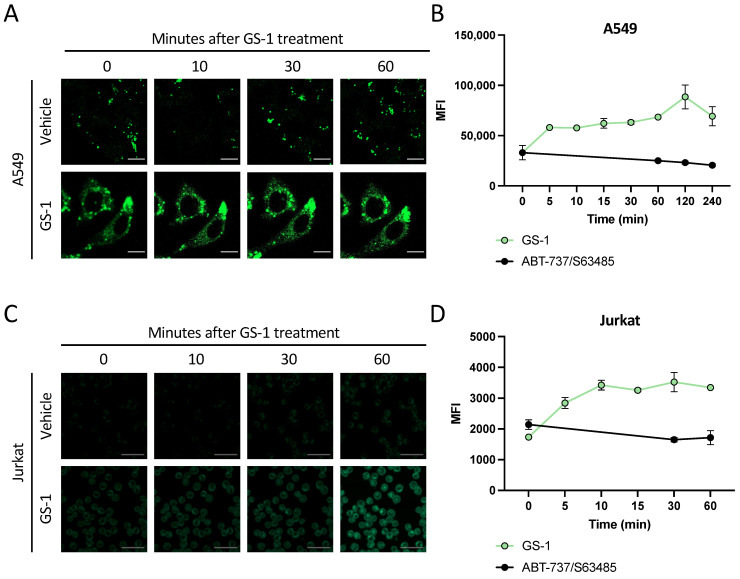

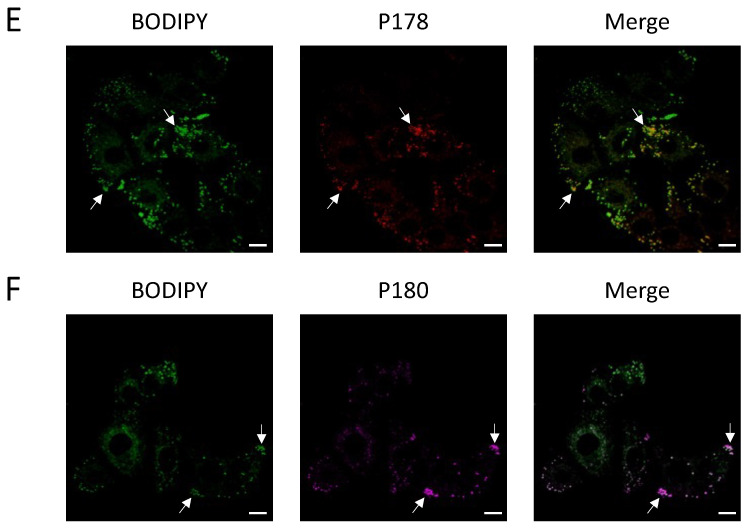
GS-1 localizes to lipid droplets. (**A**) Confocal time course microscopy analysis of A549 cells stained with BODIPY and treated with GS-1 (scale bar = 10 µm; images representative of 3 experiments). (**B**) A549 cells were stained with BODIPY and treated with GS-1 prior to analysis by flow cytometry to determine lipid droplet levels as indicated by BODIPY MFI (n = 3, ±S.E.M.). (**C**) Confocal time course microscopy analysis of Jurkat cells stained with BODIPY and treated with GS-1 (scale bar 50 µm; images representative of 3 experiments). (**D**) Jurkat cells were stained with BODIPY and treated with GS-1 prior to analysis by flow cytometry to determine lipid droplet levels as indicated by BODIPY MFI (n = 3, ±S.E.M.). A549 cells were stained with BODIPY and either (**E**) Dye 1 or (**F**) Dye 2 for 4 h prior to confocal microscopy to determine GS-1 colocalization with lipid droplets (scale bars 10 µm; images representative of 3 experiments; arrows represent areas of colocalization).

When fatty acids are taken up by cells they localize to lipid droplets [6]. To understand whether the undecylenic acid in GS-1 also localized here, we used a BODIPY dye which binds neutrally charged lipids within lipid droplets. We stained cells with BODIPY and treated them with GS-1 before conducting confocal microscopy. The addition of GS-1 rapidly increased punctate BODIPY staining within lipid droplets (and some cytoplasmic staining) within 10 min of treatment in A549 (Figure 4A) and Jurkat (Figure 4C) cells compared to vehicle controls. This rapid increase in BODIPY staining upon GS-1 treatment was also observed in flow cytometry analysis of A549 (Figure 4B) and Jurkat (Figure 4D) cells.

To confirm that undecylenic acid localized to lipid droplets, we generated two fluorescently labelled undecylenic acid probes, Dye 1 and Dye 2. BODIPY-stained A549 cells were treated with either Dye and imaged via confocal microscopy. Both Dye 1 and 2 demonstrated clear colocalization with BODIPY (Figure 4E,F). Notably, there was also some cytoplasmic staining with Dye 1 and 2 suggesting some free undecylenic acid in cells in addition to lipid droplet accumulation (Figure 4E,F) in agreement with data in Figure 4A. These results suggest that undecylenic acid in GS-1 is rapidly taken up by cells and like other fatty acids localize to lipid droplets.

### 2.6. FATP2 Likely Plays a Role in GS-1 Uptake

**Figure 5:**
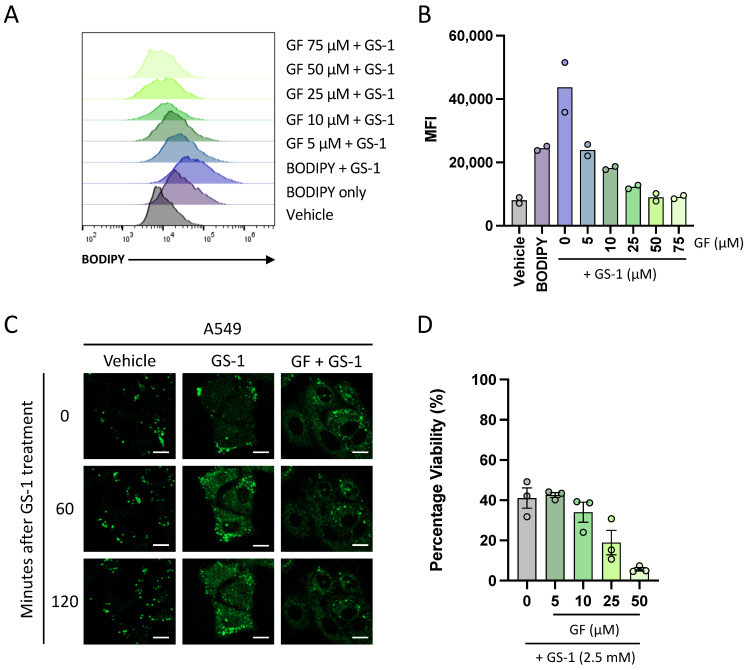
GS-1 likely enters cells via FATP2. (**A**) A549 cells were stained with BODIPY and treated with GS-1 and increasing concentrations of Grassofermata for 4 h. BODIPY levels were plotted as histograms after flow cytometry analysis. BODIPY MFI was then quantified in (**B**) (Representative of n = 3 experiments). (**C**) A549 cells were stained with BODIPY and treated with GS-1 with or without Grassofermata (50 µM; images representative of 3 experiments) and imaged every 15 min for 2 h (scale bar 10 µm). (**D**) A549 cells were treated with GS-1 and increasing concentrations of Grassofermata for 24 h before undergoing an MTT assay to determine viability (n = 3, ±S.E.M.).

Once we established GS-1 localization to lipid droplets, we then sought to understand how GS-1 was taken up by cells. FATP2 is a part of the Fatty Acid Transport Protein family normally expressed in the liver and kidney. However, it is also upregulated in tumor cells including the A549 cell line [29,30,31,32]. To investigate the role of FATP2 in GS-1 uptake we treated BODIPY-stained A549 cells with Grassofermata, a non-competitive inhibitor of FATP2 [31], before subjecting them to flow cytometry analysis. Interestingly, increasing concentrations of Grassofermata correlated with reduced BODIPY fluorescence following GS-1 treatment, indicating FATP2 blockade resulted in less intracellular GS-1 (Figure 5A,B). To confirm this, we conducted fluorescence confocal microscopy on BODIPY-stained A549 cells in the presence of Grassofermata. Similarly, the FATP2 inhibitor was able to significantly reduce BODIPY staining after GS-1 treatment (Figure 5C), suggesting less intracellular GS-1. Taken together, these data demonstrated that inhibiting FATP2 decreased intracellular lipid droplet levels, suggesting a role for FATP2 in GS-1 uptake.

With a role for FATP2 now established in GS-1 uptake, we hypothesized that internalization via this channel would be important for GS-1 cytotoxicity. To test this, viability assays were performed with GS-1 in the presence of the FATP2 inhibitor. Unexpectedly, increasing concentrations of Grassofermata correlated with increased GS-1 cytotoxicity (Figure 5D). These data suggest that GS-1 toxicity may not be entirely dependent on FATP2 or intracellular uptake.

### 2.7. Blocking Lipid Droplet Formation May Not Affect GS-1 Toxicity

**Figure 6:**
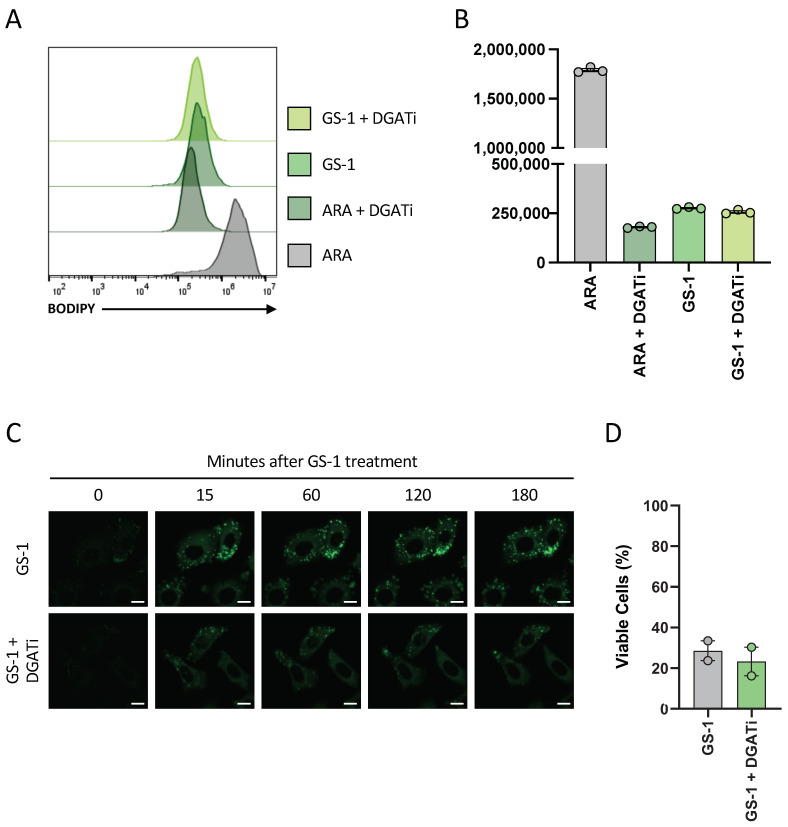
Role of LD in GS-1 cytotoxicity. A549 cells were treated with GS-1 and DGATi for 4 h before being stained with BODIPY. (**A**) BODIPY levels were plotted as histograms after flow cytometry analysis. BODIPY MFI was then quantified in (**B**) (Representative of n = 3 experiments, ±S.E.M). (**C**) Confocal time course microscopy analysis of A549 cells treated with GS-1 in the presence of DGATi and stained with BODIPY (scale bar 10 µm; images representative of 3 experiments). (**D**) A549 cells were treated GS-1 with or without DGATi for 24 h before undergoing an MTT assay to determine viability (n = 2 independent experiments, ±S.E.M).

Excess lipids are sequestered into lipid droplets to protect cells from fatty acid oxidation [6,33]. Our observation that GS-1 localized to lipid droplets (Figure 4) led us to hypothesize that cells sequester undecylenic acid in lipid droplets upon GS-1 treatment to protect themselves from fatty acid-mediated toxicities. To test this, prior to GS-1 treatment, we inhibited DGAT1 and DGAT2 with DGAT inhibitors (DGATi) which are essential for the biogenesis of new lipid droplets. Interestingly, inhibiting lipid droplet formation with DGATi did not reduce BODIPY fluorescence in GS-1-treated cells as expected (Figure 6A,B). The DGAT inhibitors were indeed blocking new lipid droplet formation; performing the experiment with a different fatty acid, arachidonic acid, resulted in a significantly lower BODIPY signal in the presence of DGATi (Figure 6A,B). These results were replicated in live cell imaging where a time-dependent increase in BODIPY staining after GS-1 treatment was nonetheless observed despite lipid droplet inhibition (Figure 6C). It should be noted that BODIPY staining in DGATi-treated cells was visibly less than in GS-1-only treated cells, suggesting some discrepancy between the qualitative and quantitative data. However, DGAT inhibition was also not able to exacerbate GS-1-mediated toxicity (Figure 6D). Therefore, these data together suggest that lipid droplet biogenesis and undecylenic acid sequestration to lipid droplets may not play a protective role in GS-1-induced cell death.

## 3. Discussion

The MUFA undecylenic acid has previously been shown to have antifungal properties, however, applications outside of this are yet to be explored. Other fatty acids have been shown to have tumoricidal properties against a myriad of tumor cell lines [4,5,6,7,8,10,11,12]. Both PUFAs and SFAs have been shown to induce tumor cell death [6,7,8,10,11,12,13,14,17], however, the precise molecular basis has yet to be fully elucidated, with many cell death pathways implicated in different studies [18]. One of the main challenges when investigating fatty acids as potential treatment options is their limited solubility in water. Due to their hydrophobic nature, often lipophilic solvents have to be used which comes with the potential of added off-target toxicities [34] and limitations on the concentrations able to be used.

In this study, we established a novel platform for the solubility of fatty acids and showed that undecylenic acid, in the form of GS-1, has pro-apoptotic tumoricidal activity. GS compounds use an amino acid to solubilize fatty acids, as detailed in this paper for GS-1 where L-arginine promotes undecylenic acid solubility in water. This mechanism circumvents the current need to use toxic lipophilic solvents or chemical modifications that can reduce activity. In addition, we have been able to show that GS-1-mediated cell death is reliant on pro-apoptotic proteins like caspase-3 and -7 and causes a loss of mitochondrial membrane potential. This expands on previous knowledge of the anti-cancer nature of other PUFAs and SFAs and contributes to understanding the molecular nature of fatty acid-induced tumor cell death, in particular for MUFAs like undecylenic acid. Notably, this study describes some of the first in vitro interactions of undecylenic acid with human cells. We have demonstrated that exogenous undecylenic acid, in the form of GS-1, localizes to lipid droplets after entering the cell via the fatty acid transport channel FATP2. However, unlike other fatty acids, lipid droplets did not play a protective role in GS-1-mediated cytotoxicity. This work describes for the first time the pro-apoptotic nature of undecylenic acid and identifies a new strategy for the solubilization of fatty acids to be used in a clinical setting.

In this study we describe GS-1, a novel formulation for the solubilization of undecylenic acid using L-arginine. The supramolecular structures of GS-1 shown in this study suggest that the hydrophobic tails of the fatty acid pack together, shielded from the polar water molecules by the amino acid in solution, and this packing of each unit is what confers water solubility of GS-1. There are many benefits of using amino acids for solubility. As fatty acids are weakly soluble in water, most previous studies have used lipophilic solvents such as ethanol [4,5,8,9,11,17,35] or DMSO which have been associated with toxicities during treatment [34]. The use of amino acids reduces possible toxicities associated with these solvents, making GS compounds suitable for clinical use. Importantly, this body of work has also demonstrated that this platform for solubility allows fatty acids to retain their active cytotoxic properties, as demonstrated with undecylenic acid here, making this approach even more viable as a possible treatment option. Furthermore, as amino acids are a part of the human diet, this allows for GS compounds to be used at much higher concentrations than other small-molecule anti-cancer therapies. Moreover, the high concentrations of GS-1 used here are likely to be safe as previous work has shown that undecylenic acid is well tolerated in humans. Most commonly, undecylenic acid is used topically as a metal salt conjugate (e.g., zinc). In this form, undecylenic acid is available clinically at concentrations up to 25% w/w. Moreover, acknowledging that GS-1 is also toxic to non-cancerous human cells, GS-1 is not tumor-cell-specific. However, as preliminary toxicology studies suggest that GS-1 is tolerated and safe at doses that exceed therapeutic exposure, GS-1 is still viable as a potential treatment option. Based on these studies, such high concentrations and exposure time, such as in the HUVEC viability assay, would not be physiologically relevant and unlikely to occur in in vivo settings, thereby the in vivo toxicology data is more reliable in predicting GS-1 safety profile than in vitro studies. However, overall, the novel solubility platform described here is likely to produce anti-cancer fatty acids that are likely safe and retain efficacy for use as a potential anti-cancer treatment option.

Previously the only known application for undecylenic acid was as an antifungal agent. This work expands on this knowledge of undecylenic acid and presents the first evidence of a pro-apoptotic anti-cancer mechanism of action. Undecylenic acid is effective in inhibiting biofilm formation and reducing transcriptional regulation of the cell membrane virulence factors and cell proliferation in *Candida albicans* [3]. Here, we have shown that undecylenic acid is also capable of inducing tumor cell death in both adherent and suspension human tumor cell lines. Previously it has been shown that a lack of Stearoyl-CoA desaturase 1 and MUFA synthesis can induce tumor cell death, through both cell cycle arrest or the induction of and UPR [36,37,38]. However, the data generated here is the first evidence that exogenous treatment of tumor cells with undecylenic acid is also able to induce pro-apoptotic tumor cell death. This is shown through dependence on caspase 3 and 7, exposure of phosphatidylserine and a reduction in mitochondrial membrane potential which are key characteristics of apoptotic cell death [39,40,41,42].

Other fatty acids have previously been shown to also induce apoptotic cell death. The SFA palmitate has been shown to induce apoptotic cell death in MDA-MB-231 breast cancer cells by causing a decrease in mitochondrial phospholipids like cardiolipin, altering mitochondrial function and inducing cytochrome c release and cell death [7]. Similarly, PUFAs arachidonic acid and gamma-linolenic acid are also known to cause a reduction in mitochondrial membrane potential and ATP production in LoVo colon cancer cell lines leading to mitochondrial-mediated apoptotic cell death. Hence, our research reports similar findings to previous studies and expands the knowledge of fatty acid-mediated cancer cell death to MUFAs which have not been extensively studied previously. Markedly, knowing that GS-1, and by extension undecylenic acid, can induce pro-apoptotic cell death is promising for the use of this compound in a clinical setting; apoptosis is well established to be an anti-inflammatory form of cell death, meaning in vivo cell death associated with the use of GS-1 would likely not evoke inflammation post-treatment [43,44]. However, before definitive conclusions are made further studies accounting for how apoptosis is initiated after GS-1 treatment would be beneficial before GS-1 is considered for trial in a more clinical setting.

Not only are tumor cells known to metabolize fatty acids differently from healthy cells by undergoing de novo lipogenesis to meet their lipid demands [45], they also possess mechanisms to protect themselves from fatty acid-induced cell death. One such protective mechanism is lipid droplet formation. Lipid droplets are suggested to protect tumor cells against the development of reactive oxygen species (ROS) and oxidative stress [33]. Exogenous fatty acids are known to localize to lipid droplets [4,5,6]. Consistent with this previous work, we have shown here that undecylenic acid and GS-1 alike localize to lipid droplets (Figure 4). This is significant as prior to this study, little was known about the interaction of undecylenic acid with human cells other than trace amounts found in human sweat. Knowing this, future work may be able to better understand how GS-1 induces apoptosis.

A previous study has demonstrated that lipid droplets played a protective role against PUFA-induced cell death as DGAT inhibitors increased the cytotoxicity of PUFAs in HCT-116 colon cancer cells [6]. Similar work done here suggested that lipid droplets do not play a protective role in GS-1-mediated cell death (Figure 6). Notably, of the studies that have previously shown lipid droplets play a protective role in fatty acid-induce cell death, the majority have investigated PUFAs. In this context, the protection lipid droplets provide to the cell makes sense as the lipid droplets are shielding the reactive double bonds from oxidative processes, preventing oxidative stress and cell death. Based on this idea, it could be postulated that GS-1, and undecylenic acid due to the presence of only a single double bond, does not induce oxidative stress as a mechanism of inducing apoptosis. Hence, GS-1 may act more like SFAs than PUFAs, which could explain why lipid droplets do not play a protective role in GS-1-mediated cell death.

In addition to establishing that undecylenic acid localizes to lipid droplets, this study has also shown for the first time that undecylenic acid may utilize FATP2 to enter cells. This adds to the knowledge of the anti-cancer properties of undecylenic acid as it suggests that it may need to be internalized to exert its cytotoxic properties. Furthermore, it is well established that FATP2 expression is upregulated in several human cancers [46] which justifies a targetable use in cancer treatment. These data are also significant as they demonstrate that the GS formulation does not affect fatty acid uptake. Fatty acids are known to bind albumin in serum, however, studies have shown that while albumin may be present, it is not directly involved in the uptake of fatty acids [47,48]. Based on this idea, it can be hypothesized that the L-arginine component of GS-1 acts like albumin, in that it allows solubility of undecylenic acid in an aqueous solution but dissociates prior to uptake.

The fatty acid transport protein (FATP) family, of which FATP2 is a member, are considered bifunctional proteins that function in both fatty acid transport and activation. FATP2, in particular, is known to have a conserved ATP/AMP motif that is involved in ATP hydrolysis as well as a FATP/VLACS motif that is responsible for fatty acid uptake [30,49,50]. Other than these motifs, little is known about the catalytic activity of FATP or their direct binding to fatty acids. This knowledge does suggest, however, that L-arginine and undecylenic acid in GS-1 dissociate prior to cell entry as it would be unlikely that an amino acid could be involved without disturbing the uptake of undecylenic acid.

One of the more interesting observations from this study was that despite FATP2 being important for GS-1 uptake, inhibition of FATP2 could not protect cells from GS-1-induced cell death. This suggests that GS-1 may utilize other mediators of fatty acid transport such as CD36, or that GS-1 could act on the plasma membrane. The fatty acid binding pocket of CD36 specifically binds the carboxyl group of fatty acids before translocating them into cells [51]. The use of CD36 for GS-1 uptake would therefore be unlikely if L-arginine and undecylenic acid were still bound due to their proposed interactions which would block the binding to CD36, furthering the hypothesis of dissociation before cell entry. A further understanding of the biochemical and physical properties of GS-1 components and whether the undecylenic acid and L-arginine components remain bound upon cell interaction, will need to be explored to gain a more robust idea of all the ways GS-1 may be entering or interacting with cells and inducing cell death.

As this novel platform for solubilizing fatty acids has few safety concerns, GS compounds have the potential to be used in a broader context and as a treatment option for other diseases, not just as an anti-cancer treatment. Undecylenic acid is already commercially available as an antifungal treatment, in a metal-salt form. Other delivery strategies include the use of hexosomes to deliver undecylenic acid to fungal infections [52]. Nevertheless, studies have reported that the use of hexosomes is limited by an inability to alter pore and channel size, small mesopores that do not allow loading of large nanoparticles as well as the need for stabilising agents [53,54]. The platform described here provides a novel and simple mechanism for the solubility of undecylenic acid to be better used in an antifungal setting. Notably, this platform allows fatty acids to retain their activity. Fatty acids are known to have a broad range of activity; it has been well established that fatty acids also have antibacterial properties and are a viable treatment option for bacterial infections [55]. Hence, in addition to antifungal applications, this platform could be used to establish other GS compounds for use as a next-generation approach to treat bacterial infections.

As for a potential application in an anti-cancer setting, future studies building upon this body of work should focus on more clinically relevant models. While this body of work provides an understanding of the molecular mechanisms of how GS-1 induces tumor cell apoptosis in vitro it cannot provide insight into the efficacy of GS-1 in an in vivo setting. Testing of GS-1 in animal models of cancer will give great insight into the activity of undecylenic acid in GS-1 in vivo.

Past research has shown that the tumoricidal nature of fatty acids is underutilized in the fight against cancer. The novel platform described here, where GS compounds use an amino acid to solubilize fatty acids, provides a new and safe mechanism for fatty acids to be studied and applied in an anti-cancer setting. In addition, this work identifies a pro-apoptotic mechanism of GS-1-mediated tumor cell death, providing a better understanding of the action of undecylenic acid and fatty acids in general, that contributes to the re-emerging field of fatty acids as potential anti-cancer therapeutics.

## 4. Materials and Methods

### 4.1. Cell Culture

All culture media was supplemented with 10% (*v*/*v*) FBS (Bovogen, New Zealand), 0.2% (*v*/*v*), MycoZap (Lonza, Basel, CH, Switzerland), 100 U/mL penicillin and 100 U/mL streptomycin (Thermo Fisher Scientific) unless otherwise stated, and from here on will be referred to as complete media. HeLa, U937, Jurkat, A549, Cas9 and caspase 3/7 double knock out (DKO) U937 cells were cultured in RPMI (Gibco) (all cell lines were purchases from ATCC unless otherwise stated). Wild type (WT) and Bax/Bak DKO HTC-116 cells were cultured in DMEM (Life Technologies, New York, NY, USA). Human Umbilical Vein Endothelial cells (HUVEC) were cultured in EGM-2 (Lonza). U937 media was supplemented with 5% FBS. A549, HeLa, Jurkat and U937 cells were cultured at 37 °C and 5% CO_2_ and HCT-116 cells were cultured at 37 °C with 10% CO_2_.

### 4.2. Transmission Electron Microscopy (TEM)

Copper TEM grids with a formvar-carbon support film (GSCU300CC-50, ProSciTech, Qld, Australia) were glow discharged for 60 s in an Emitech k950x with k350 attachment. GS-1 was diluted in water to 0.01 mM, 0.1 mM, 0.8 mM, and 1 mM. Four microlitre drops of GS-1 suspension was pipetted onto each grid, allowed to adsorb for at least 30 s and blotted with filter paper. Two drops of 2% uranyl acetate were used to negatively stain the particles blotting after 10 s each. Grids were then allowed to dry before imaging. Grids were imaged using a Joel JEM-2100 (JEOL (Australasia) Pty Ltd., Brookvale, Australia) transmission electron microscope equipped with a Gatan Orius SC 200 CCD camera (Scitek Australia).

### 4.3. Nuclear Magnetic Resonance (NMR)

An aliquot (250 μL) of GS-1 was diluted with 20 mL MilliQ water, adjusted to pH 4, and extracted 3 times with 40 mL heptane. The aqueous fraction was freeze-dried and reconstituted with D2O for ^1^H and ^13^C analysis. The combined heptane fractions were dried under vacuum and taken into CDCl_3_ for ^1^H and ^13^C analysis.

### 4.4. Gas Chromatography-Mass Spectrometry (GCMS)

GS-1 was analyzed as a mixture, followed by analysis of both aqueous and heptane-extracted fractions. All detected peaks were matched against the National Institute of Standards and Technology (NIST) database for annotation. Following whole formulation analysis, 250 μL of GS-1 was diluted with 20 mL MilliQ water and extracted 3 times with 40 mL heptane. The aqueous fraction was freeze-dried, derivatized with n,o-bis (trimethylsilyl)trifluoroacetamide and resuspended in methanol for trace analysis. The combined heptane fractions were dried under vacuum, resuspended in n-hexane and analyzed separately.

### 4.5. Viability Assays

HeLa, A549, Jurkat, U937, HUVEC caspase 3/7 double knock out (DKO) and Cas9 U937 cells (a kind gift from Professor Christine Hawkins, La Trobe University, Bundoora, Australia), as well as Bax/Bak DKO and WT HCT-116 cells (a kind gift from Professor Hamsa Puthalakath, La Trobe University), were seeded into 96-well plates at 1 × 104 cells per well in complete media. Adherent cells were seeded the day before allowing for adherence. Cells were treated with increasing concentrations of GS-1 and undecylenic acid as well as 4.3 mM L-arginine. Wells containing complete medium alone and cells alone were included in triplicate as a background and vehicle control, respectively. For adherent cells, after 24 h incubation, cell viability was determined using 3-(4,5-dimethylthiazol-2-yl)-2,5-diphenyl tetrazolium bromide (MTT). Briefly, MTT (1 mg/mL) was added to each well (100 μL) and plates were incubated for 2 h (37 °C and 5% CO_2_). After incubation, media was removed and replaced with dimethyl sulfoxide (100 μL, DMSO, Sigma-Aldrich, Burlington, MA, USA) and placed on a plate shaker for 5 min to dissolve tetrazolium salts, after which absorbance was measured at 570 nm. For suspension cells 3-(4,5-dimethylthiazol-2-yl)-5-(3-carboxymethoxyphenyl)-2-(4-sufophenyl)-2H-tetrazolium (MTS) (Merck, Darmstadt, Germany) and phenazine methosulfate (PMS) (Merck, Darmstadt, Germany) were used to assess viability. Briefly, an MTS (2 mg/mL) and PMS solution was added to wells (20 μL) and the plate was incubated for 2 h (37 °C and 5% CO_2_) after which absorbance was measured at 490 nm. The absorbance of the vehicle control was designated as 100% viability; all values were corrected according to the background control reading.

### 4.6. Flow Cytometry Viability Assay

A549 and Jurkat cells were seeded in 96-well plates at a density of 2 × 104 and 1.5 × 105 cells/well, respectively, in 1% Bovine Serum Albumin (BSA) (Sigma-Aldrich) RPMI. A549 cells were incubated overnight to allow for adherence. Cells were then treated with vehicle or increasing concentrations of GS-1 and incubated for 3 h (Jurkats) or 6 h (A549 s) at 37 °C and 5% CO_2_. BH3 mimetics were used as a positive control; Jurkats were treated with 1.25 μM ABT-737 (Selleck Chemicals, Houston, TX, USA) and 0.125 μM S63845 (Selleck Chemicals, TX, USA). A549 s were treated with 5 μM ABT-737 and 2 μM S63845. After incubation, cells were stained with Annexin-A5-FITC (AV) (BD Biosciences, San Jose, CA, USA) and To-Pro-3-APC (Life Technologies) in 1× AV Binding Buffer (BD Biosciences, San Jose, CA, USA) and incubated for 10 min on ice before being analyzed and gated using a gating strategy from Jiang et al. (2016). Analysis was performed on a CytoFLEX S flow cytometer (Beckman Coulter). Flow cytometric data were analyzed with FlowJo software (Tree Star, version 10.8, Ashland, OR, USA).

### 4.7. Confocal Laser Scanning Microscopy

Live cell imaging was performed on a Zeiss 800 Confocal Laser Scanning Microscope (Zeiss, Germany) at the La Trobe Bioimaging Platform using 63× oil immersion at 37 °C and 5% CO_2_. All cells were imaged in an 8-well chamber (Nunc, Denmark) and prepared using 1% BSA RPMI. A549 cells (2 × 10^4^ cells/well) were seeded the day before imaging to allow for adherence. Jurkat cells (1.5 × 10^5^ cells/well) were immobilized using 10% poly-L-lysine coated chambers. In all experiments, GS-1 was prepared at 2.7 mM for A549 s or 2 mM for Jurkats. Stains including MitoTracker Red (Thermo Fisher Scientific, MA) and 4,4-Difluoro-1,3,5,7,8-Pentamethyl-4-Bora-3a,4a-Diaza-s-Indacene (BODIPY) (0.1 mg/mL) (Thermo Fisher Scientific, Waltham, MA, USA), were prepared and added to cells prior to imaging. In certain experiments cells were treated with inhibitors including 50 µM Grassofermata (inhibitor of FATP2) (Sapphire Bioscience, NSW, AUS) or Diglyceride acyltransferase inhibitor 1 (DGATi1; 2 mM) and Diglyceride acyltransferase inhibitor (DGATi2; 10 mM) (kind gifts from Professor Karla Helbig; collectively referred to as DGATi), inhibitors of lipid droplet biogenesis, prior to imaging. Cells were imaged at time points stated in figure legends. Images were analyzed using Zen Image Analysis software (Zen Blue Edition version 10.1.19043, Jena, Germany).

### 4.8. Western Blot

A549 cells were seeded into a 6-well plate in complete media at a density of 5 × 10^5^ cells per well and incubated overnight (37 °C and 5% CO_2_). Jurkat cells were seeded into a 12-well plate in complete media at a density of 1 × 10^6^ cells per well. Both cell types were treated with GS-1 as well as vehicle control and a BH3 mimetic-treated positive control (5 µM ABT-737 and 2 µM S63845). Following treatment, cells were harvested and lysed immediately in PMN lysis buffer (1% Nonidet P40, 10% glycerol, 1% TX-100, 3% NaCl 5 M, 2% HEPES 1 M) with protease inhibitor on ice via rigorous resuspension and lysates were collected. Cell lysate protein concentration was estimated using the Pierce Bicinchoninic acid (BCA) Protein Assay Kit (Thermo Scientific, Rockford, IL, USA) according to the manufacturer’s instructions. Following protein quantification, lysates (20 µg protein) were incubated with NuPAGE LDS (lithium dodecyl sulphate) sample buffer (Invitrogen) and 1 M dithiothreitol. Samples were separated on a 4–12% Bis-Tris gel (Thermo Fisher Scientific) in NuPAGE MES running buffer (Life Technologies) using SeeBlue Plus 2 Protein Ladder (Invitrogen). Proteins were transferred onto a polyvinylidene fluoride (PVDF) membrane.

Post transfer, membranes were subsequently blocked and washed before being incubated with rabbit-anti-pro-caspase-3 (1:1000 in 1% BSA PBST) (Santa Cruz, H-277 lot #12013) overnight at 4 °C. Membranes were washed and incubated with goat anti-rabbit-HRP (1:5000, 0.1% PBTS with 5% skim milk powder) (Thermo Fisher) for 1 h at room temperature. This process was then repeated with rabbit-anti-cleaved-caspase-3 (Cell Signalling, D175 lot #43). Mouse-anti-ß-actin (1:1000 in 1% BSA PBST) (Sigma-Aldrich, cat# A2228, clone#AC-74) was used as a loading control, using sheep-anti-mouse HRP (1:5000, 0.1% PBTS with 5% skim milk powder) (GE Healthcare) secondary antibody. Membranes were imaged using ECL prime detection reagent (Bio-strategy) and the Syngene gel documentation system (Syngene, Frederick, MD, USA).

### 4.9. BODIPY Uptake Assay

A549 and Jurkat cells were seeded in 96-well plates at a density of 2 × 10^4^ and 1.5 × 10^5^ cells/well, respectively, in 1% BSA RPMI. A549 cells were incubated (37 °C and 5% CO_2_) overnight to allow for adherence. Cytochalasin-D (CytoD) (Sigma Aldrich) an inhibitor of actin polymerization, was used to prevent cell disassembly during cell death. BODIPY (0.1 mg/mL), Cytochalasin-D (CytoD) (5 mM) (Sigma Aldrich) and GS-1 (2.7 mM) were prepared in 1% BSA RPMI. GS-1 was added to cells at time points stated in figure legends prior to analysis of BODIPY fluorescence. Before analysis, A549 cells were washed (30 µL PBS) and lifted using 30 µL Trypsin (Life Technologies, Carlsbad, CA, USA) and subsequently neutralized with 70 µL complete media before all being transferred to a second U-bottom plate. After centrifugation A549 s were re-suspended in complete 1% BSA RPMI. Analysis was performed on a CytoFLEX S flow cytometer (Beckman Coulter, Singapore). Flow cytometric data were analyzed with FlowJo software (Tree Star, version 10.8).

### 4.10. Synthesis of Fluorescent Undecylenic Acid Probes

Unless otherwise stated, all reactions were performed with reagent-grade solvents. All reagents and solvents were obtained from commercial sources and used without further purification. Reactions were monitored using thin-layer chromatography (TLC) and visualized using UV light and stained using a basic KMnO_4_ (potassium permanganate) solution. Flash column chromatography was performed using a Biotage^®^ Isolera TM on Biotage^®^ KP-Sil SNAP cartridges. Petrol refers to petroleum spirit (b.p. 40–60 °C). NMR spectra (^1^H and ^13^C) were recorded on Bruker Ascend TM 400 (400 MHz) spectrometer as dilute solutions in the stipulated solvent. All chemical shifts (δ) are reported in parts per million (ppm) with 1H and 13C NMR referenced to solvent signals [ 1H NMR: CDCl_3_ (7.27), DMSO-*d_6_* (2.50); 13C NMR: CDCl_3_ (77.16), DMSO-*d_6_* (39.52)]. Coupling constants (J) are reported in Hertz (Hz) and recorded after averaging. The multiplicity of the 1H NMR signals are designated by one of the following abbreviations: s = singlet, d = doublet, t = triplet, q = quartet, m = multiplet, appt s = apparent singlet, appt d = apparent doublet, appt t = apparent triplet signal. HRMS spectra were acquired by using a Thermo Scientific Q Exactive Plus Orbitrap LC–MS/MS instrument operating in ESI (Electrospray ionization) mode 4-(1,2,2-triphenylvinyl)aniline was synthesized according to literature procedures [56].

#### 4.10.1. Synthesis of Dye 1

**Scheme 1:**
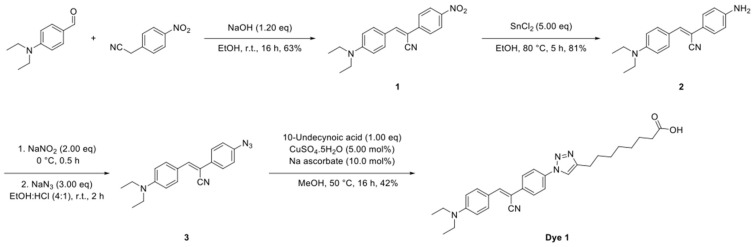
Synthetic scheme for probe 178 (Dye 1).

The synthetic route to Dye 1 is shown in Scheme 1 and the detailed synthesis procedures and structural characterization are shown as following.

Synthesis of (Z)-3-(4-(Diethylamino)phenyl)-2-(4-nitrophenyl)acrylonitrile (**1**)

To a solution of 4-(diethylamino)benzaldehyde (885 mg, 5.00 mmol, 1.00 eq) and 2-(4-iodophenyl)acetonitrile (810 mg, 5.00 mmol, 1.00 eq) in EtOH (100 mL) was added NaOH (240 mg, 6.00 mmol, 1.20 eq) and the solution stirred at room temperature for 16 h. The reaction mixture was cooled to 0 °C, the resultant precipitate was collected by filtration and dried under a vacuum to give the title compound (1) as a purple solid (1.01 g, 63%). **^1^H NMR** (400 MHz, CDCl_3_) δ 8.28−8.18 (m, 2 H), 7.94−7.86 (m, 2 H), 7.79−7.71 (m, 2 H), 7.52 (s, 1 H), 6.71 (d, *J* = 9.1 Hz, 2 H), 3.45 (q, *J* = 7.1 Hz, 4 H), 1.23 (t, J = 7.1 Hz, 6 H). **^13^C NMR** (101 MHz, CDCl_3_) δ 146.9, 145.4, 142.3, 132.7, 125.7, 124.4, 119.0, 111.8, 45.0, 12.7 (Supplementary Figures S6 and S7).

Synthesis of (Z)-2-(4-Aminophenyl)-3-(4-(diethylamino)phenyl)acrylonitrile (**2**)

To a solution of (*Z*)-3-(4-(diethylamino)phenyl)-2-(4-nitrophenyl)acrylonitrile (**1**) (873 mg, 3.00 mmol, 1.00 eq) in EtOH (50.0 mL) was added SnCl_2_ (2.84 g, 15.0 mmol, 5.00 eq) and the solution stirred at 80 °C for 5 h. The reaction mixture was cooled to room temperature, filtered through a pad of Celite^®^ and concentrated under reduced pressure. The crude residue was redissolved in EtOAc (50.0 mL) and washed with water (50.0 mL). The organic phase was dried over MgSO_4_, filtered and the solvent removed under reduced pressure to give the title compound (2) as a yellow solid (707 mg, 81%). **^1^H NMR** (400 MHz, CDCl_3_) δ 7.78 (d, *J* = 9.0 Hz, 2 H), 7.43 (d, *J* = 8.5 Hz, 2 H), 7.22 (s, 1 H), 6.75 − 6.63 (m, 4 H), 3.78 (s, 2 H), 3.42 (q, *J* = 7.0 Hz, 4 H), 1.20 (t, *J* = 7.0 Hz, 6 H). **^13^C NMR** (101 MHz, DMSO-d_6_) δ 149.0, 148.4, 137.7, 130.6, 125.9, 122.0, 120.9, 119.7, 114.0, 111.0, 103.2, 43.7, 12.5 (Supplementary Figures S8 and S9).

Synthesis of (Z)-2-(4-Azidophenyl)-3-(4-(diethylamino)phenyl)acrylonitrile (**3**)

To a solution of (*Z*)-2-(4-aminophenyl)-3-(4-(diethylamino)phenyl)acrylonitrile (**2**) (159 mg, 500 µmol, 1.00 eq) in a mixture of EtOH and conc. HCl (10.0 mL, 4:1) at 0 °C was added NaNO_2_ (69.0 mg, 1.00 mmol, 2.00 eq). The reaction mixture was stirred at 0 °C for 0.5 h followed by the portion wise addition of NaN_3_ (98.0 mg, 1.50 mmol, 3.00 eq). The resultant solution was warmed to room temperature and stirred for 2 h, diluted with EtOAc (20.0 mL) and washed with brine (20.0 mL). The organic phase was dried over MgSO_4_, filtered and concentrated under reduced pressure and the crude product used without further purification.

Synthesis of (Z)-8-(1-(4-(1-Cyano-2-(4-(diethylamino)phenyl)vinyl)phenyl)-1H-1,2,3-triazol-4-yl)octanoic Acid (Dye 1)

To a solution of the crude (*Z*)-2-(4-azidophenyl)-3-(4-(diethylamino)phenyl)acrylonitrile (**3**) and 10-undecynoic acid (91.0 mg, 500 µmol, 1.00 eq) in MeOH (5.00 mL) was added CuSO_4_.5H_2_O (6.00 mg, 25.0 µmol, 5.00 mol%) and Na ascorbate (10.0 mg, 50.0 µmol 10.0 mol%) and the solution stirred at 50 °C for 16 h. The reaction mixture was diluted with H_2_O (15.0 mL) and extracted into EtOAc (2 × 15.0 mL). The organic phases were combined, dried over MgSO_4_, filtered and concentrated under reduced pressure. The crude residue was purified by flash column chromatography (0–10% MeOH in CH_2_Cl_2_) to give the title compound (Dye 1) as a light brown solid (102 mg, 42% over two steps). **^1^H NMR** (400 MHz, DMSO-*d_6_*) δ 11.96 (s, 1 H), 8.63 (s, 1 H), 7.97 (d, *J* = 8.4 Hz, 2 H), 7.90 (s, 1 H), 7.88−7.84 (m, 3 H), 6.80 (d, *J* = 8.9 Hz, 2 H), 3.44 (q, *J* = 6.8 Hz, 4 H), 2.73−2.67 (m, 2 H), 2.16−2.11 (m, 2 H), 1.71−1.64 (m, 2 H), 1.50 (s, 4 H), 1.36−1.30 (m, 6 H), 1.14 (t, *J* = 6.8 Hz, 6 H). **^13^C NMR** (101 MHz, DMSO-*d_6_*) δ 149.5, 143.5, 135.8, 134.8, 131.8, 126.1, 120.1, 120.0, 111.1, 100.2, 84.5, 71.1, 43.8, 28.8, 28.7, 28.6, 28.3, 28.1, 27.9, 25.0, 17.6, 12.5. HRMS (ESI^+^): calculated for C_29_H_34_N_5_O_2_ [M-H+]: m/z = 484.2718, m/z found 484.2710 (Supplementary Figures S10 and S11).

#### 4.10.2. Synthesis of Dye 2 

**Scheme 2:**
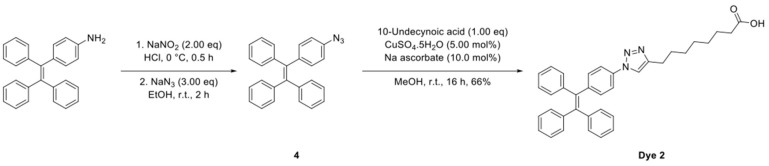
Synthetic scheme for Dye 2 (probe 180).

The synthetic route to Dye 2 is shown in Scheme 2 and the detailed synthesis procedures and structural characterization are shown as following.

Synthesis of (2-(4-Azidophenyl)ethene-1,1,2-triyl)tribenzene (**4**)

To a solution of 4-(1,2,2-triphenylvinyl)aniline (174 mg, 500 µmol, 1.00 eq) in a mixture of EtOH and conc. HCl (10.0 mL, 4:1) at 0 °C was added NaNO_2_ (69.0 mg, 1.00 mmol, 2.00 eq). The reaction mixture was stirred at 0 °C for 0.5 h followed by the small addition of NaN_3_ (98.0 mg, 1.50 mmol, 3.00 eq). The resultant solution was warmed to room temperature and stirred for 2 h, diluted with EtOAc (20.0 mL) and washed with brine (20.0 mL). The organic phase was dried over MgSO_4_, filtered and concentrated under reduced pressure and the crude product used without further purification.

8-(1-(4-(1,2,2-Triphenylvinyl)phenyl)-1H-1,2,3-triazol-4-yl)octanoic Acid (Dye 2)

To a solution of the crude (2-(4-azidophenyl)ethene-1,1,2-triyl)tribenzene (**4**) and 10-undecynoic acid (91.0 mg, 500 µmol, 1.00 eq) in MeOH (5.00 mL) was added CuSO_4_.5H_2_O (6.00 mg, 25.0 µmol, 5.00 mol%) and Na ascorbate (10.0 mg, 50.0 µmol 10.0 mol%) and the solution stirred at 50 °C for 16 h. The reaction mixture was diluted with H_2_O (15.0 mL) and extracted into EtOAc (2 × 15.0 mL). The organic phases were combined, dried over MgSO_4_, filtered and concentrated under reduced pressure. The crude residue was purified by flash column chromatography (0–10% MeOH in CH_2_Cl_2_) to give the title compound (Dye 2) as an off white solid (179 mg, 66% over two steps). **^1^H NMR** (400 MHz, CDCl_3_) δ 7.48 (br s, 2 H), 7.17 (d, *J* = 7.6 Hz, 2 H), 7.15−7.09 (m, 10 H), 7.08−6.99 (m, 6 H), 2.78 (br s, 1 H), 2.35 (t, *J* = 7.4 Hz, 2 H), 1.73 (s, 2 H), 1.65−1.60 (m, 2 H), 1.37−1.31 (m, 7 H). **^13^C NMR** (101 MHz, CDCl_3_) 179.4, 179.3, 144.5, 143.4, 143.3, 143.2, 142.3, 139.6, 135.3, 133.0, 131.4, 131.4, 131.3, 128.5, 128.1, 128.0, 127.8, 127.0, 126.9, 126.8, 125.1, 120.1, 84.8, 68.2, 34.1, 29.2, 29.2, 29.1, 29.0, 28.8, 28.5, 24.8, 18.5. HRMS (ESI^+^): calculated for C36H34N3O2 [M-H^+^]: m/z = 540.2656, m/z found 540.2659 (Supplementary Figures S12 and S13).

### 4.11. Confocal Microscopy: Undecylenic Acid Probes Microscopy

A549 cells were seeded at a density of 2 × 10^4^ cells/well the day before to allow for adherence in an 8-well chamber (Nunc, Denmark). Cells were stained with 0.05 µg/mL BODIPY and Dye 1 or Dye 2 (250 μM) in 1% BSA RPMI and incubated for 4 h (37 °C and 5% CO_2_). After incubation, endpoint images were taken. Imaging was performed on the Zeiss 780 Confocal Laser Scanning Microscope (Zeiss, Germany) at the La Trobe Bioimaging Platform using 63× magnification with oil immersion at 37 °C and 5% CO_2_. Spectral imaging-linear unmixing was performed on the Zeiss 780 Confocal Laser Scanning Microscope to separate the fluorescent emission of BODIPY, Dye 1 and Dye 2. Images were analyzed using Zen Image Analysis software (Zen Blue Edition version 10.1.19043).

### 4.12. Uptake Analysis with FATP2 Inhibitors

A549 cells were seeded at a density of 2 × 10^4^ cells per well in a 96-well plate in complete media and incubated (37 °C and 5% CO_2_) overnight to allow for adherence. BODIPY (0.1 mg/mL), CytoD (5 mM) and Grassofermata (inhibitor of FATP2) (5, 10, 25, 50, 75 mM) were prepared in complete media. Cells were treated with a CytoD/BODIPY master mix and incubated for 5 min. Following incubation, cells were then treated with increasing concentrations of Grassofermata and again incubated for 5 min before treating with GS-1 (2.7 mM) and incubated for 4 h. Treatments were performed in duplicate with vehicle and BH3 mimetic-treated (ABT-737 at 5 μM and S63845 at 2 μM) controls included. After incubation and prior to analysis, A549 cells were lifted as described previously in ‘BODIPY uptake assay’ before being stained with BV605 A5 (BD Biosciences) and To-Pro-3-APC in 1× AV Binding Buffer and incubated for 10 min on ice. Analysis was performed on a CytoFLEX S flow cytometer (Beckman Coulter). Flow cytometric data were analyzed with FlowJo software (Tree Star, version 10.8).

### 4.13. Viability Assays with FATP2 Inhibition

Viability assays were performed as described in Section 4.5, in the presence of 5, 10, 25, or 50 µM Grassofermata to determine the role of FATP2 in GS-1 (2.7 mM)-induced cytotoxicity.

### 4.14. Involvement of Lipid Droplets in GS-1 Cytotoxicity Using DGAT Inhibition

A549 cells were seeded at a density of 2 × 10^4^ cells per well in a 96-well plate in complete media and incubated (37 °C and 5% CO_2_) overnight to allow for adherence. CytoD (5 mM), DGATi1 (2 mM), DGATi2 (10 mM) and GS-1 (2.5 mM) were prepared in complete media. All samples were treated with CytoD to prevent disassembly. A549 cells were treated with GS-1 alone or GS-1 in the presence of DGATi then incubated overnight. The next day all samples were stained with BODIPY (0.1 mg/mL) before analysis to determine lipid droplet levels. Prior to analysis, A54 cells were lifted as described previously in ‘BODIPY uptake assay’ Analysis was performed on a CytoFLEX S flow cytometer. Flow cytometric data were analyzed with FlowJo software (Tree Star, version 10.8).

### 4.15. Viability Assays with DGAT Inhibition

Viability assays were performed as described in Section 4.5, in the presence of DGATi1 (2 mM) and DGATi2 (10 mM) to determine the role of lipid droplets in GS-1 (2.5 mM) cytotoxicity.

### 4.16. Single Ascending Dose Toxicology Study with GS-1

All animal handling and treatment was approved by the University of Melbourne Animal Ethics Committee. Fifteen male Sprague Dawley rats were given a subcutaneous (between the shoulder blades) bolus dose of either 10 mL/kg saline or 10 mL/kg GS-1 at either 190.5 mg/kg (at 19.05 mg/mL), 381 mg/kg (at 38.1 mg/mL), or 762 mg/kg (at 76.2 mg/mL). Rats were observed for any adverse events or abnormal behaviour. Animals were euthanized after 24 h.

## 5. Conclusions

In conclusion, the novel formulation of undecylenic acid with arginine in GS-1 provides a solution to overcome challenges with fatty acid solubility. GS-1 is capable of mediating apoptotic cell death in a range of cancer cell lines. This is underpinned by reliance on caspases, a reduction in mitochondrial membrane potential and the presentation of typical apoptotic morphology. This study provides some of the first evidence of the tumoricidal nature of undecylenic acid in vitro and contributes to understanding the molecular mechanism by which fatty acids induce cell death.

## 6. Patents

US patent 11,103,475; Issue date 31 August 2021. Therapeutic Compositions of Undecylenic Acid and Arginine.

## Data Availability

Not applicable.

## Supplementary Materials

The following supporting information can be downloaded at: https://www.mdpi.com/article/10.3390/ijms232214170/s1.
