# Supplementary material for: Novel Formulation of Undecylenic Acid induces Tumor Cell Apoptosis
Source: Int J Mol Sci. 2022 Nov 16;23(22):14170. doi: 10.3390/ijms232214170 (PMC9692760; doi:10.3390/ijms232214170)
Supplement: Supplementary file 1 [file ijms-23-14170-s001.zip › ijms-1946258-supplementary.pdf]

A

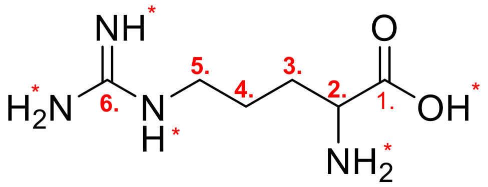

B

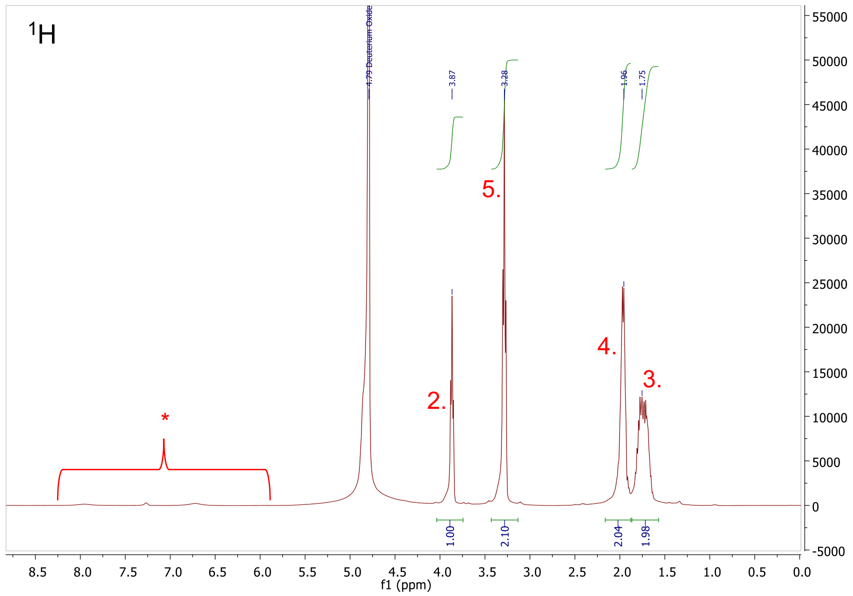

¹H NMR analysis		
Position	Expected number of protons	Found number of protons
2	1	1
3	2	1.98
4	2	2.04
5	2	2.1

C

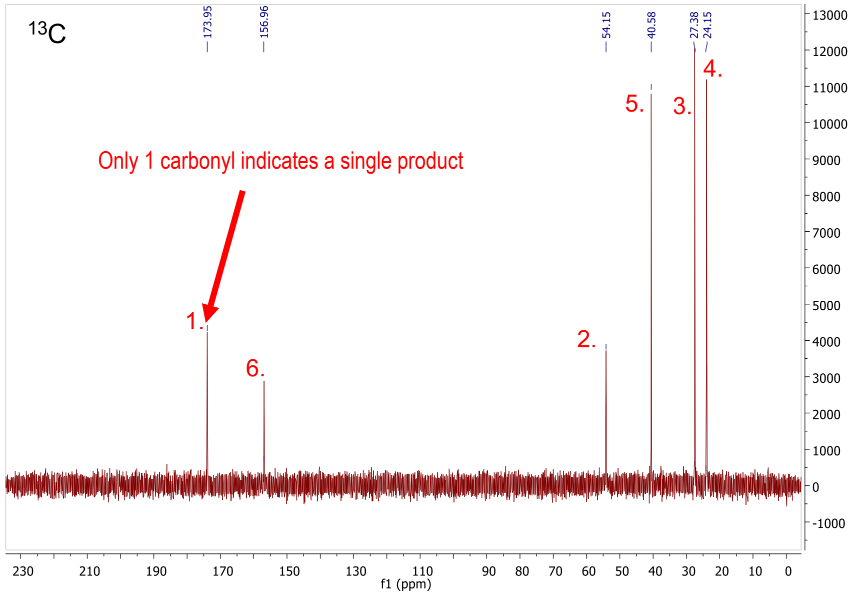

Supplementary Figure S1. (A) Schematic representation of L-arginine with carbons labeled 1–6. *These protons underrepresented in NMR plots due to fast chemical exchange. **(B)** 1H and **(C)** 13C NMR analysis of the aqueous fraction of GS-1.

A

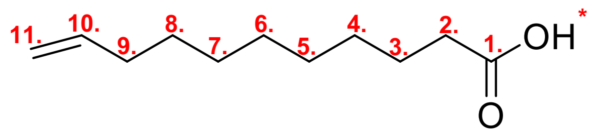

B

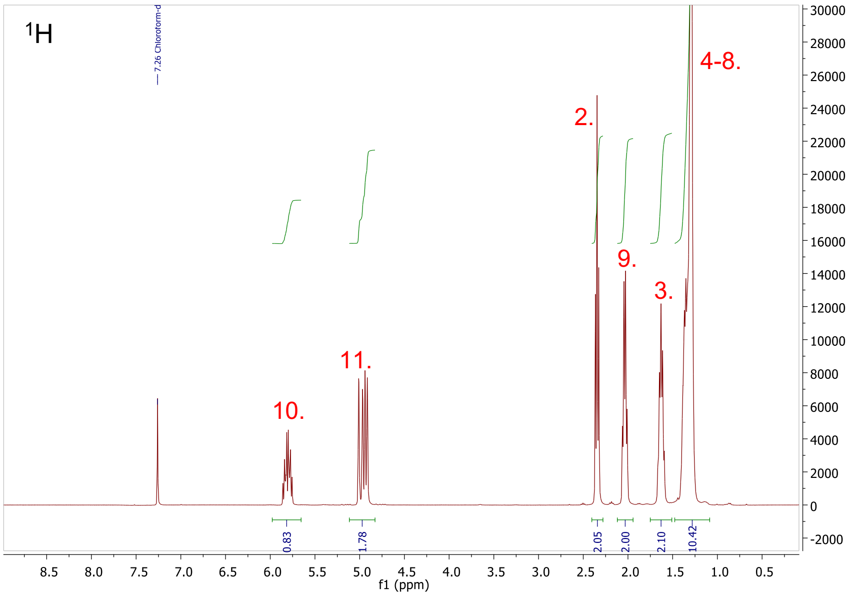

^{1}H NMR analysis		
Position	Expected number of protons	Found number of protons
2	2	2.05
3	2	2.10
4–8	10	10.42
9	2	2.0
10	1	0.83
11	2	1.78

C

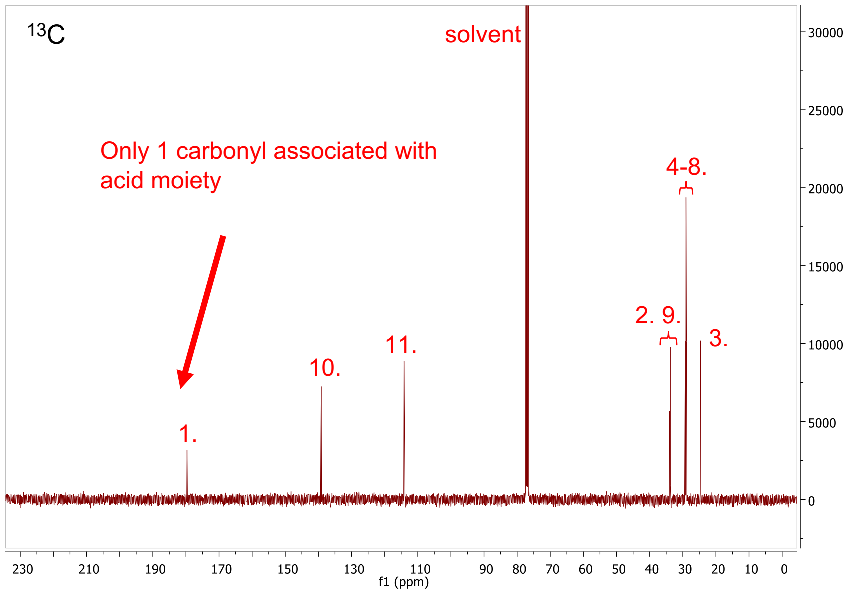

Supplementary Figure S2. (A) Schematic representation of undecylenic acid with carbons labeled 1–11. *These protons are underrepresented in NMR plots due to fast chemical exchange**. (B)** 1H and **(C)** 13C NMR analysis of the heptane fraction of GS-1..

A

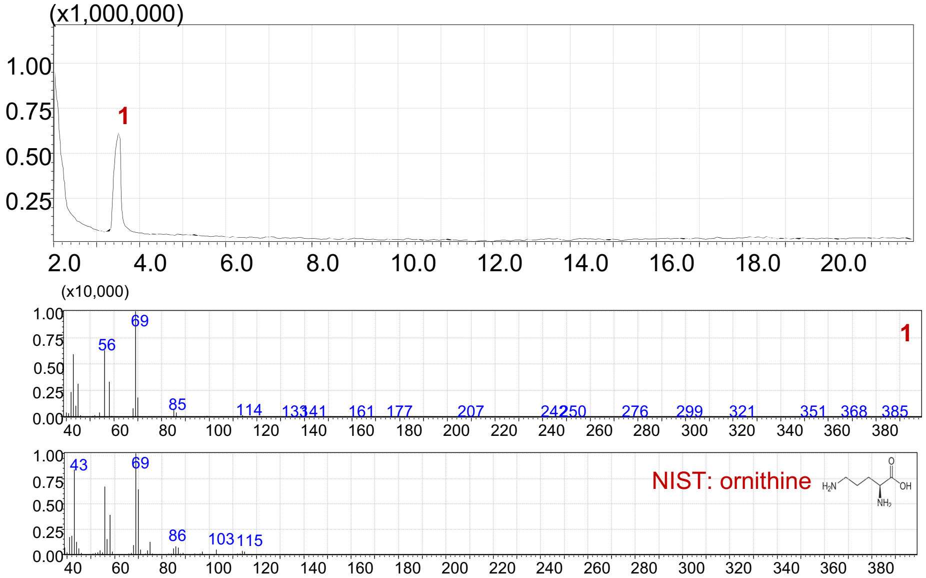

B

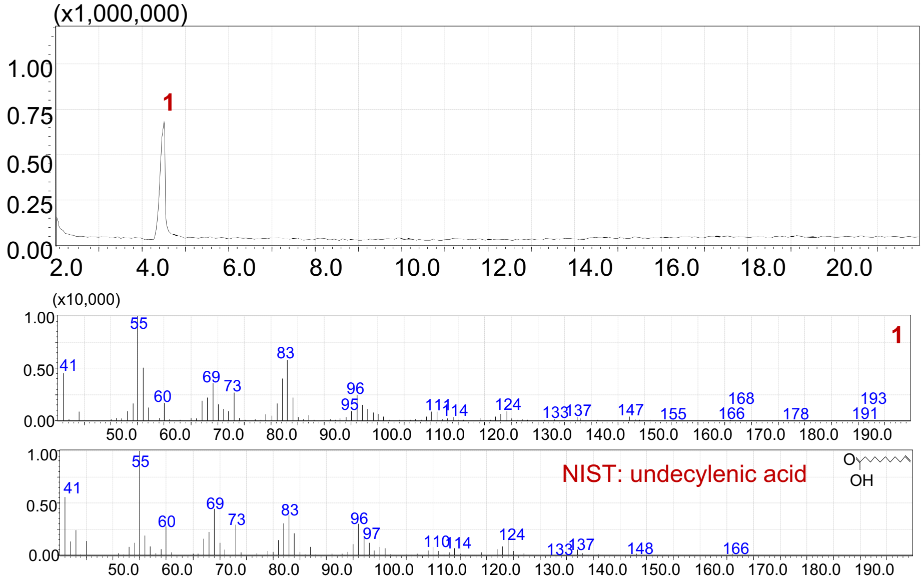

Supplementary Figure S3. GCMS of **(A)** aqueous fraction and **(B)** heptane fraction of GS-1.

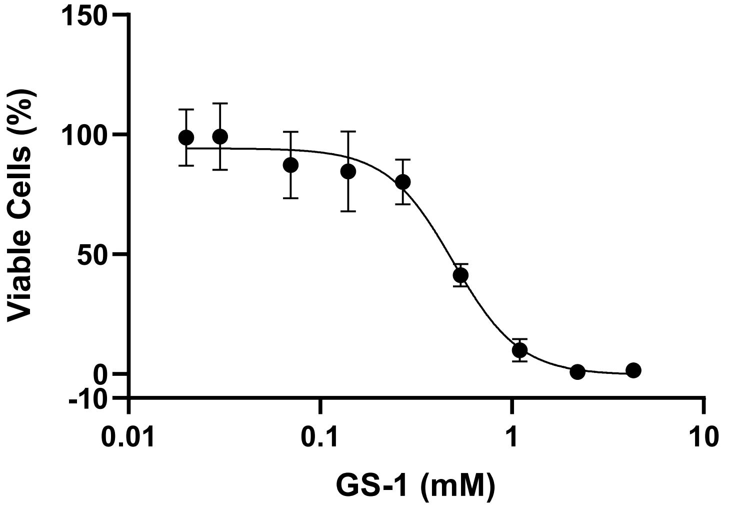

	HUVEC
IC50	0.4932

Supplementary Figure S4. HUVEC cells were treated with increasing concentrations of GS-1, for 24 h before undergoing MTT analysis to determine viability as a percentage of vehicle controls (n=3, mean ± S.E.M.).

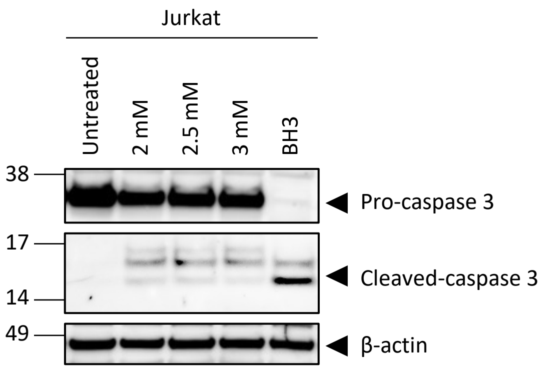

Supplementary Figure S5 Jurkat cells were treated with increasing concentrations of GS-1 for 6h before immunoblotting to determine pro-caspase 3 and cleaved-caspase 3 levels (representative of n=3 independent experiments).

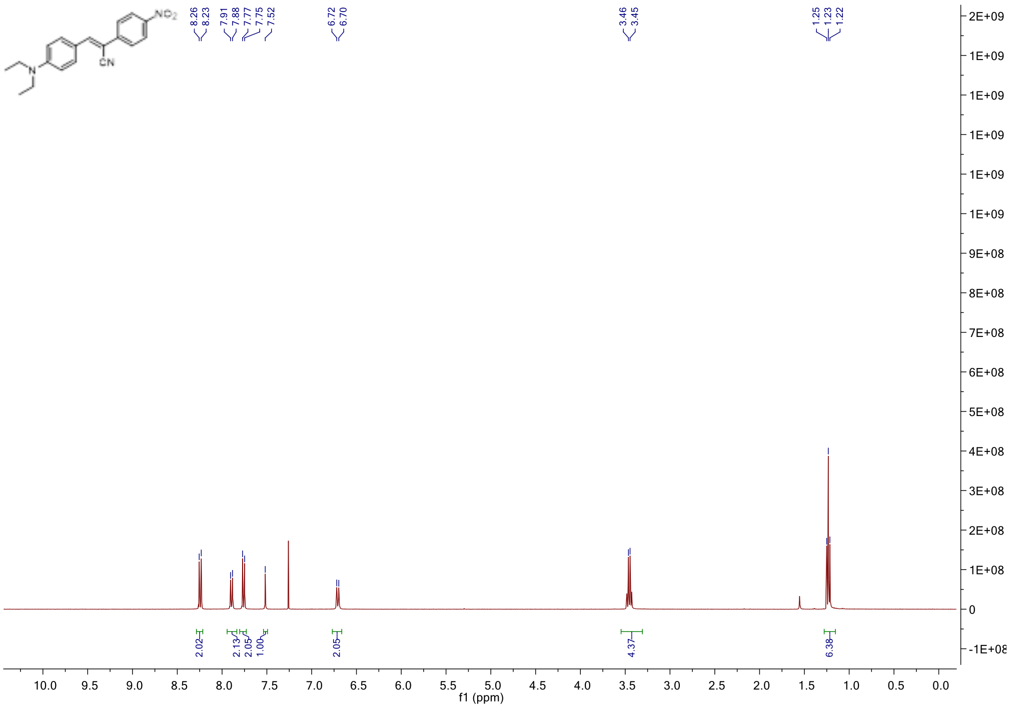

Supplementary Figure S6. ^{1}H NMR of (Z)-3-(4-(Diethylamino)phenyl)-2-(4-nitrophenyl)acrylonitrile (1).

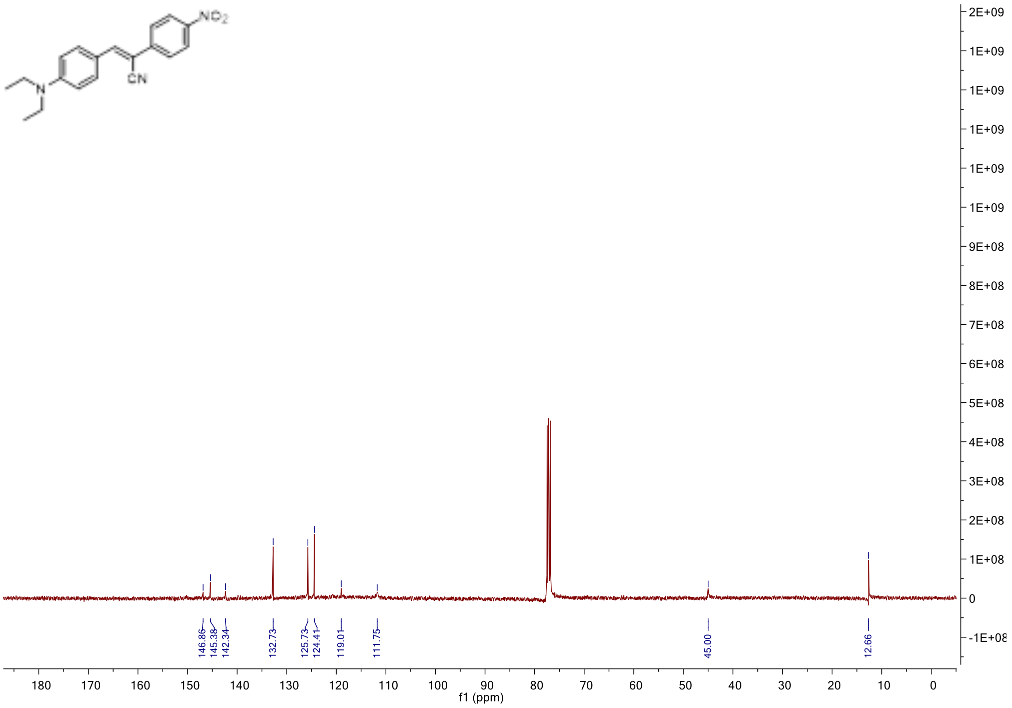

Supplementary Figure S7. ^{13}C NMR of (Z)-3-(4-(Diethylamino)phenyl)-2-(4-nitrophenyl)acrylonitrile (1).

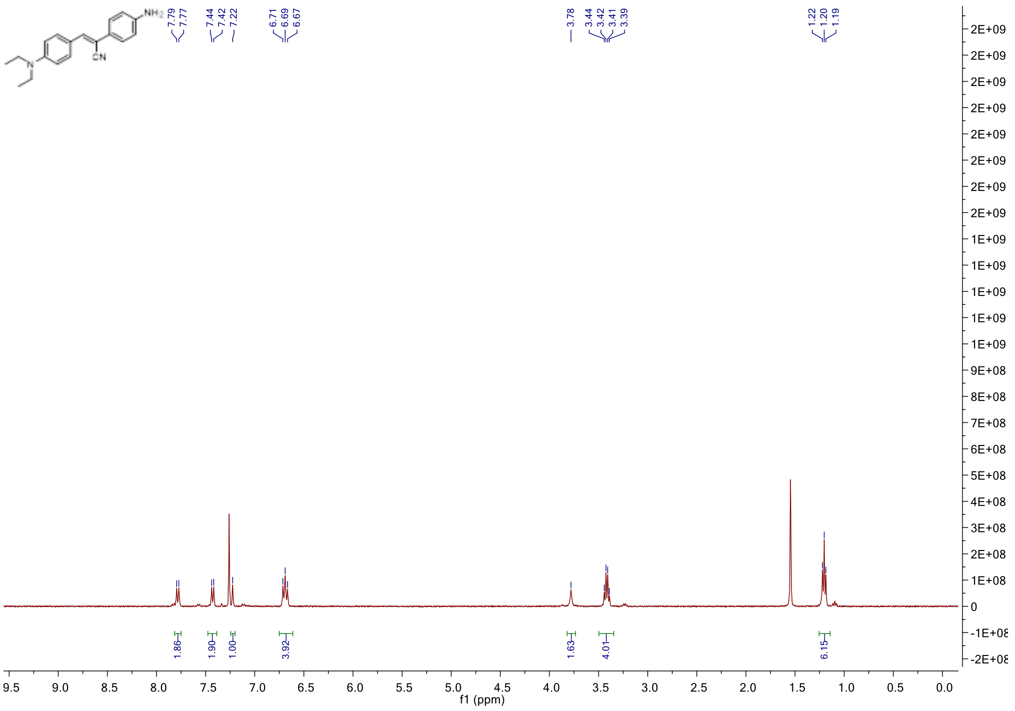

Supplementary Figure S8. ^{1}H NMR of (Z)-2-(4-Aminophenyl)-3-(4-(diethylamino)phenyl)acrylonitrile (2).

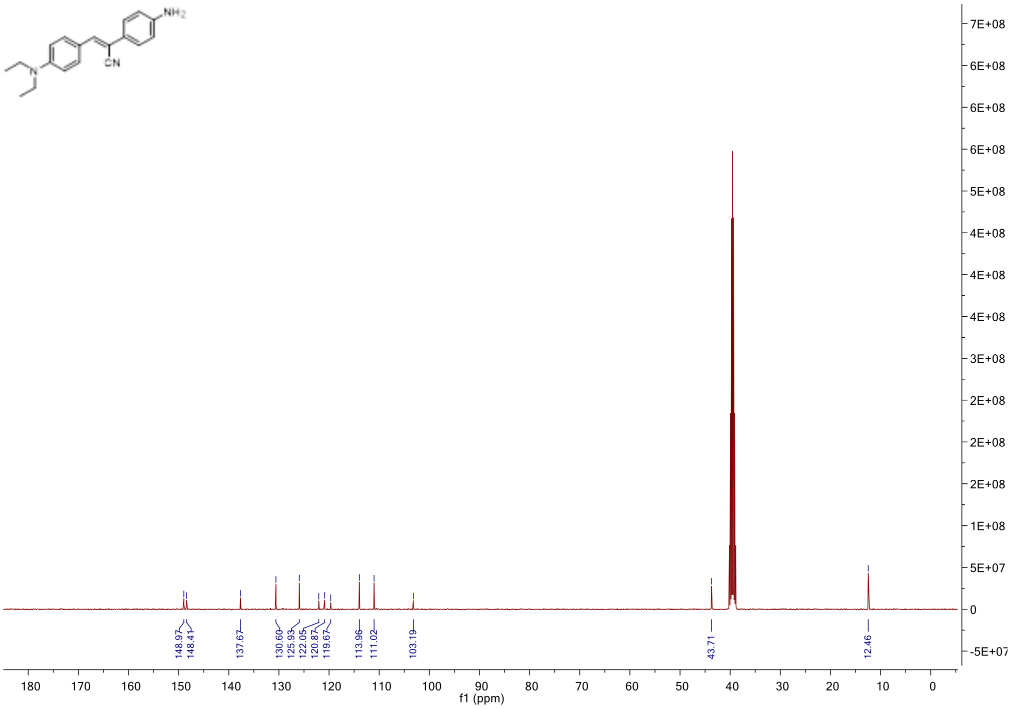

Supplementary Figure S9. ^{13}C NMR of (Z)-2-(4-Aminophenyl)-3-(4-(diethylamino)phenyl)acrylonitrile (2).

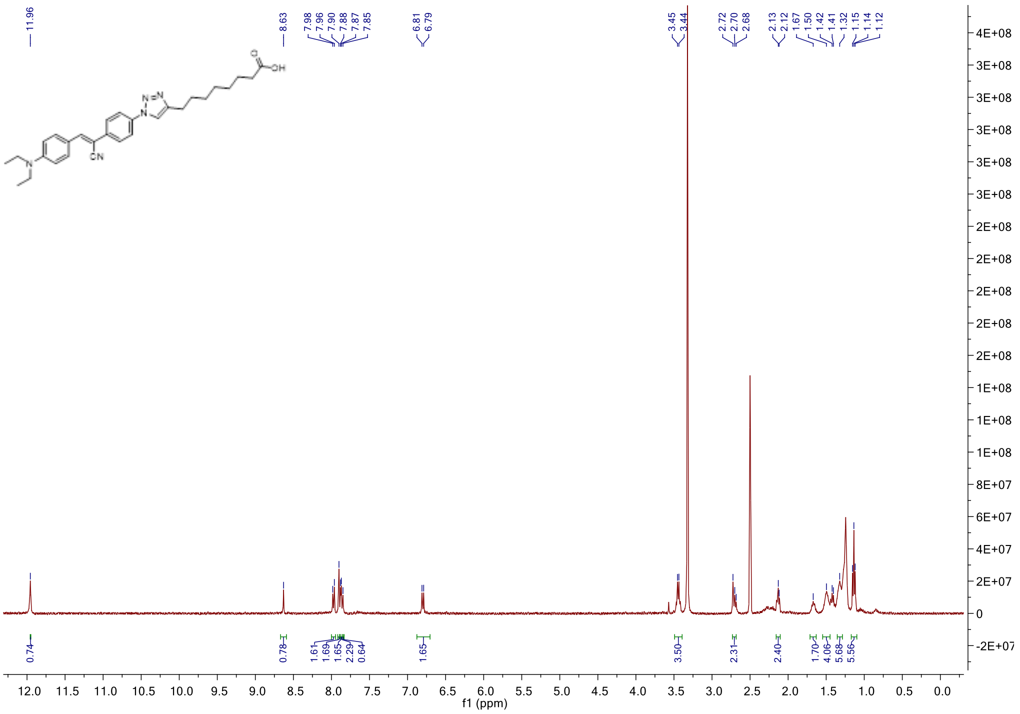

Supplementary Figure S10. ^{1}H NMR of (*Z*)-8-(1-(4-(1-Cyano-2-(4-(diethylamino)phenyl)vinyl)phenyl)-1*H*-1,2,3-triazol-4-yl)octanoic (Dye 1).

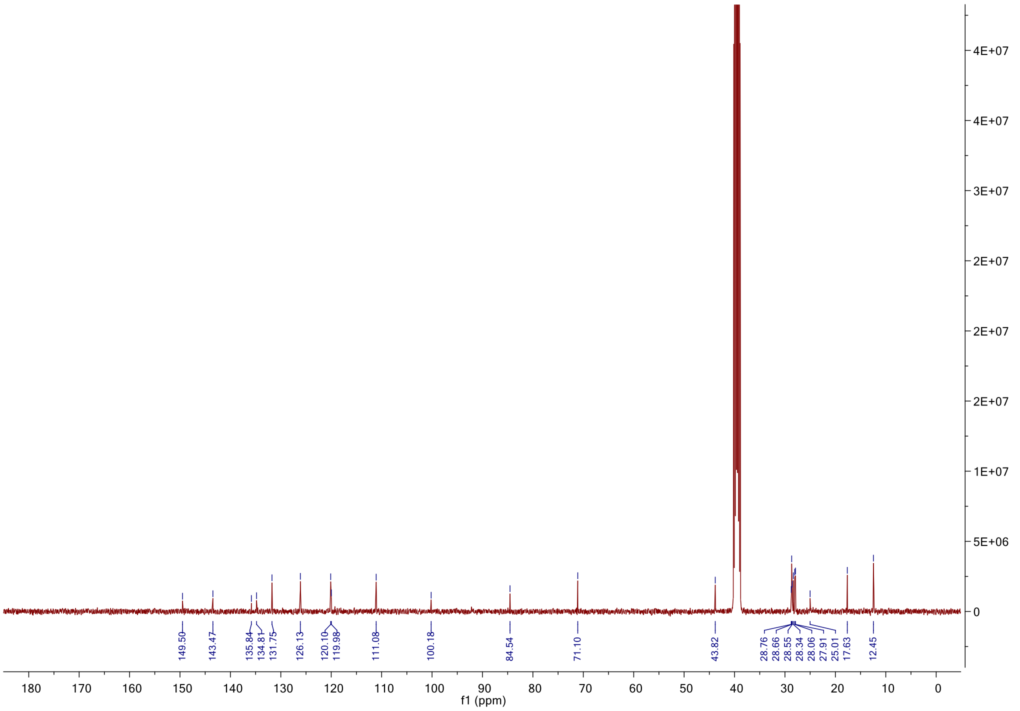

Supplementary Figure S11. ^{13}C NMR of (Z)-8-(1-(4-(1-Cyano-2-(4-(diethylamino)phenyl)vinyl)phenyl)-1H-1,2,3-triazol-4-yl)octanoic (Dye 1).

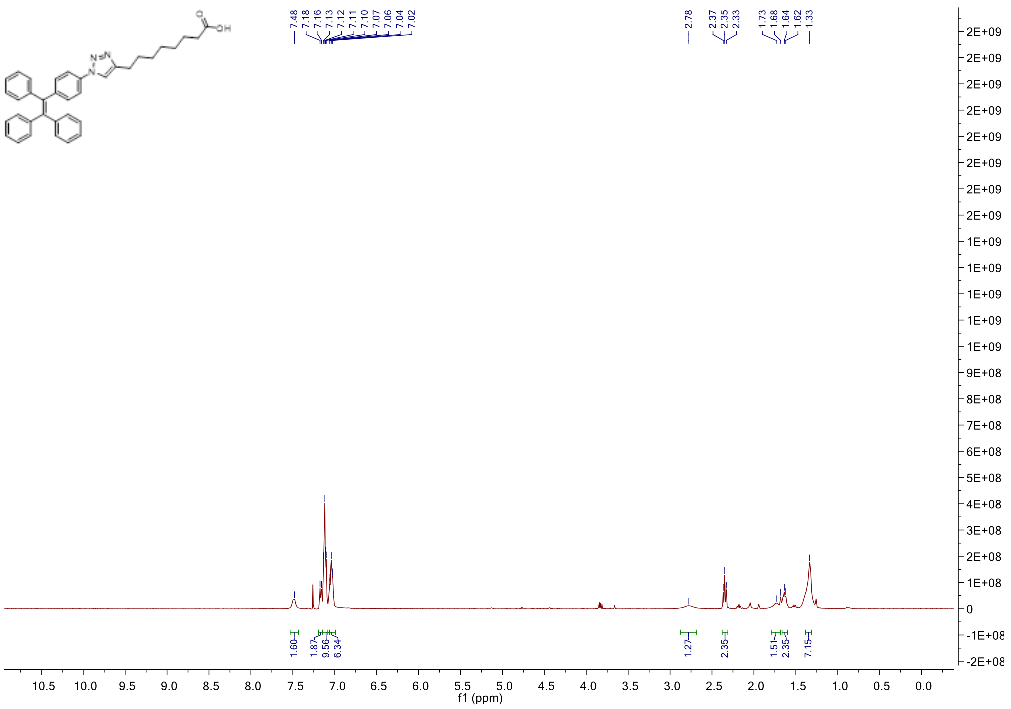

Supplementary Figure S12. · ^{1}H NMR of 8-(1-(4-(1,2,2-Triphenylvinyl)phenyl)-1H-1,2,3-triazol-4-yl)octanoic acid (Dye 2).

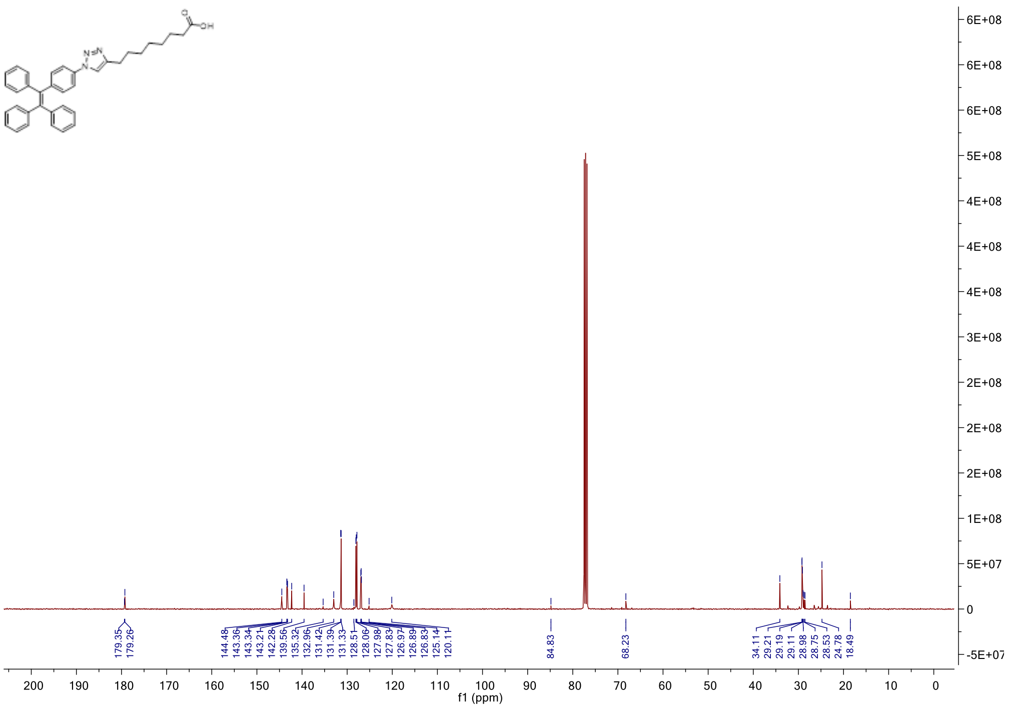

Supplementary Figure S13. ^{13}C NMR of 8-(1-(4-(1,2,2-Triphenylvinyl)phenyl)-1H-1,2,3-triazol-4-yl)octanoic acid (Dye 2).
